# Supplementary material for: The lysosomal Ragulator complex plays an essential role in leukocyte trafficking by activating myosin II
Source: Nat Commun. 2021 Jun 7;12:3333. doi: 10.1038/s41467-021-23654-3 (PMC8184920; doi:10.1038/s41467-021-23654-3)
Supplement: Supplementary file 1 — Supplementary Information [file 41467_2021_23654_MOESM1_ESM.pdf]

Supplementary Informations

The lysosomal Ragulator complex plays an essential role in leukocyte trafficking by activating myosin II

Takeshi Nakatani, Kohei Tsujimoto, JeongHoon Park,

Tatsunori Jo, Tetsuya Kimura, Yoshitomo Hayama, Hachiro Konaka,

Takayoshi Morita, Yasuhiro Kato, Masayuki Nishide,

Shyohei Koyama, Shigeyuki Nada, Masato Okada,

Hyota Takamatsu, and Atsushi Kumanogoh

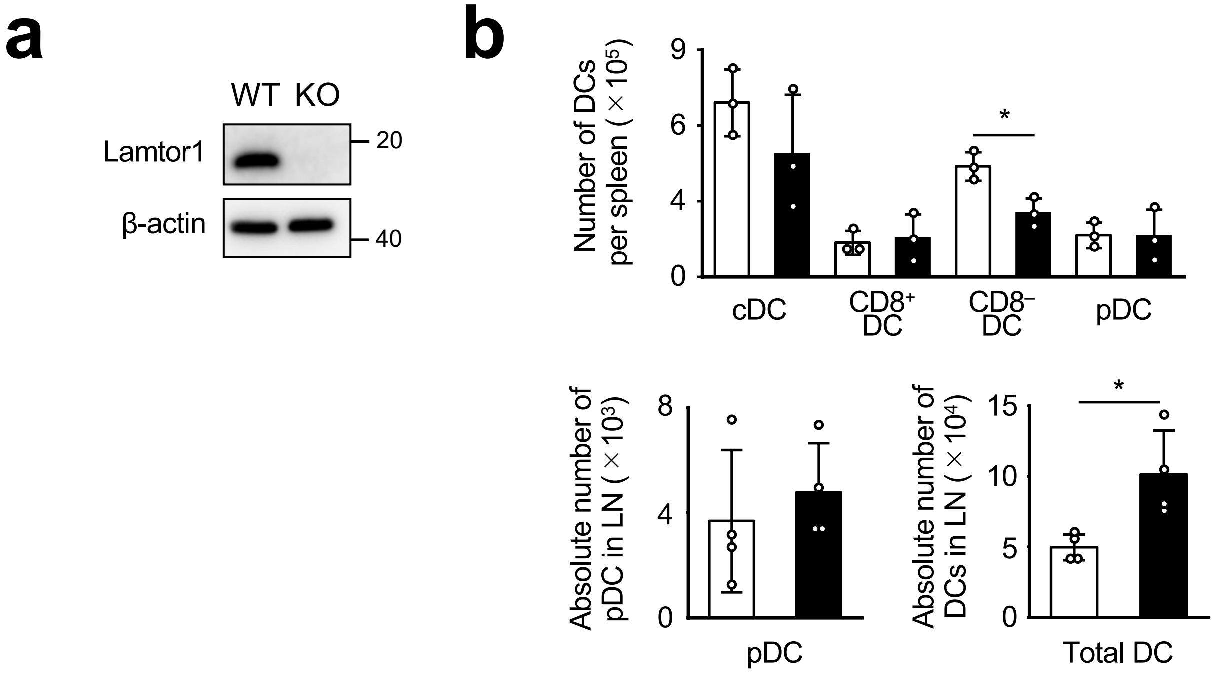

Supplementary Figure 1. Generation of DC-specific *Lamtor1*-knockout mice, and characterization of the immune cell population in CD11c-*Lamtor1*$^{-/-}$ mice.

a) Lamtor1 was not expressed in DCs isolated from CD11c-*Lamtor1*$^{-/-}$ mice. Lamtor1 protein levels in WT and *Lamtor1*$^{-/-}$ DCs are shown. Data are representative of three experiments.

b) Absolute number of the DC-subset in spleen and LNs. A single-cell suspension isolated from spleen (n=3) (upper) and inguinal LNs (n=4) (lower) of WT (white bar) and CD11c–*Lamtor1*$^{-/-}$ (black bar) mice were treated with collagenase, stained, and evaluated by flow cytometry. Conventional DCs (cDCs), CD11c^{+} I-A/I-E^{+}; CD8^{+}DC, CD11c^{+} I-A/I-E^{+} CD8^{+}; CD8-DC, CD11c^{high} I-A/I-E^{+} CD8^{-}; pDC, CD11c^{int} PDCA-1^{+}. Statistical analyses were performed by two-sided Student's t-test (**b**) [means ± s.d.; *p<0.05].

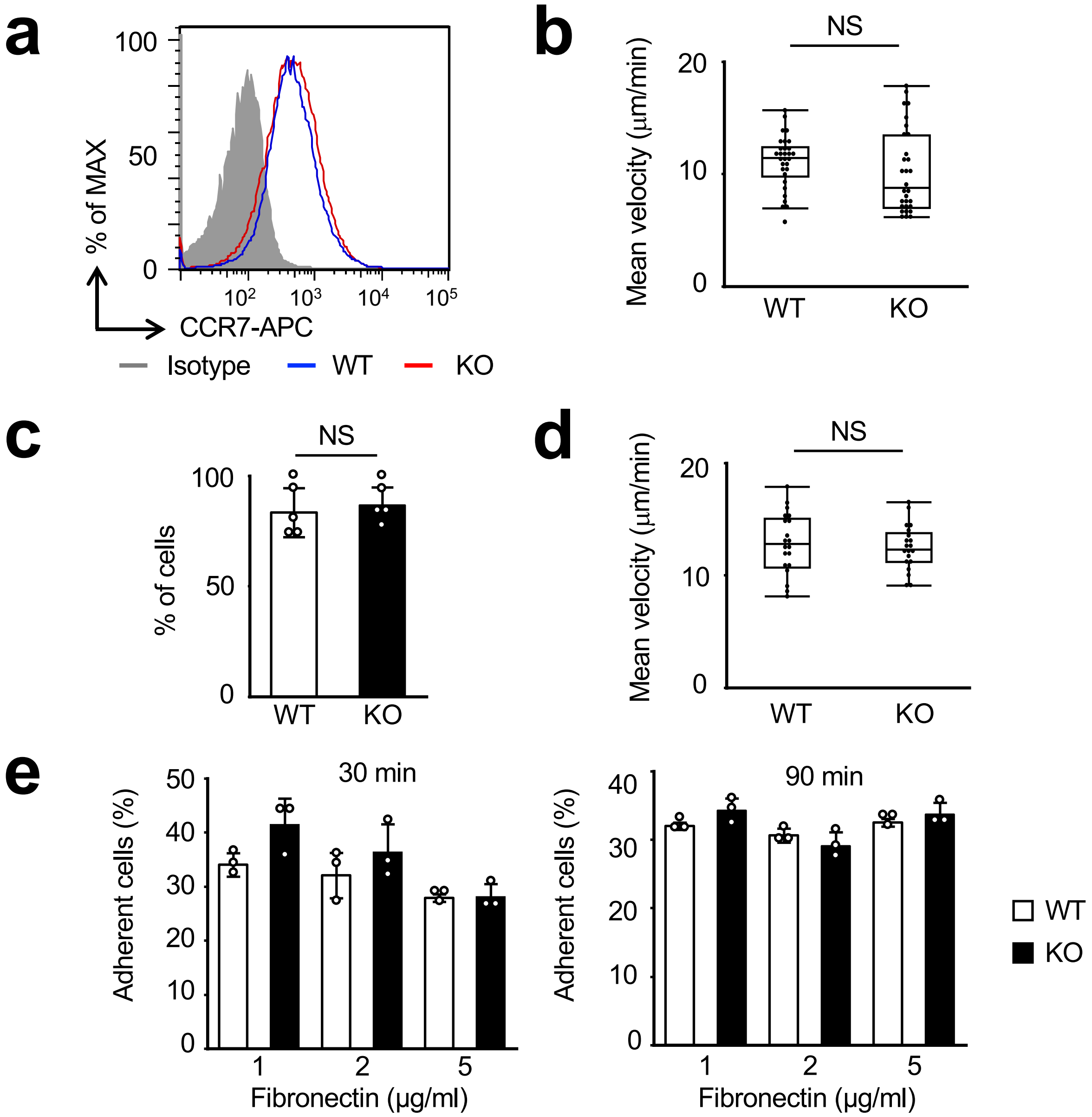

Supplementary Figure 2. DC migration in a 2D environment and adhesion of WT and *Lamtor1*$^{-/-}$ BMDCs.

a) Level of CCR7 in WT (blue line) and *Lamtor1*$^{-/-}$ (red line) BMDCs. Data are representative of three experiments.

b) Velocities of randomly moving WT and *Lamtor1*$^{-/-}$ BMDCs. Movement of DCs on coverslips coated with 10 µg/ml fibronectin was observed by time-lapse video imaging. Mean velocity of single DCs during the observation period was calculated (n=30).

c, d) Directional migration of WT (white bar) and *Lamtor1*$^{-/-}$ (black bar) BMDCs in response to CCL19. Horizontal migration of BMDCs in response to CCL19 (5 µg/ml) was visualized using an EZ-TAXIScan device (slit size, 5 µm). **c)** Directionality was assessed as DC position relative to its original position, and is presented as the percentage of cells that ended within a 30° arc facing the CCL19 source **(c)**. Mean velocity of each individual migrating DC during the observation period (n=20) **(d)**.

e) Adhesion of WT and *Lamtor1*$^{-/-}$ BMDCs to fibronectin-coated dishes. Calcein-labeled WT (white bar) and *Lamtor1*$^{-/-}$ BMDCs (black bar) were applied on the fibronectin (1, 2, or 5 µg/ml)-coated dishes for 30 or 90 min. After tight washing, cells were lysed, and fluorescence intensity was measured. Data are representative of three experiments.

Statistical analyses were performed by Student's t-test (**c, e**) [means ± s.d.; NS, not statistically significant] or two-sided Mann–Whitney U test (**b, d**) [median; 25th and 75th percentiles; and minimum and maximum of a population excluded outliers; NS, not statistically significant].

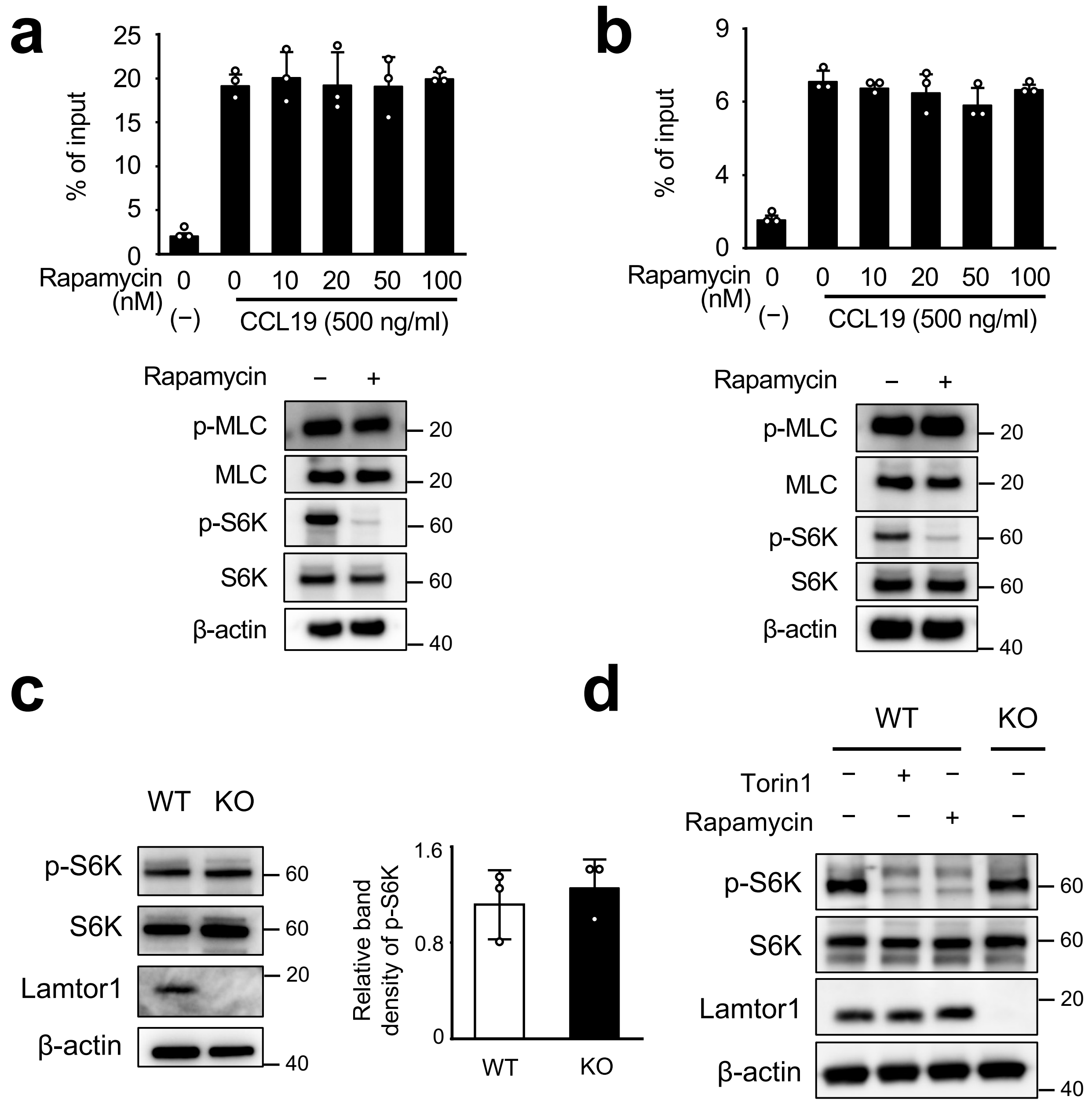

Supplementary Figure 3. S6K phosphorylation was not reduced in *Lamtor1*$^{-/-}$ DCs.

a, b) The effect of rapamycin on DC-chemotaxis in medium containing different concentrations of amino acids. Chemotaxis of WT BMDCs in response to 500 ng/ml CCL19 in the presence of different concentrations of rapamycin in DMEM medium containing high concentrations of amino acids **(a)** or RPMI-free medium containing no amino acids **(b)** was evaluated by Transwell (pore size, 5 μm) (n=3) (upper). Phosphorylation of MLC and S6K treatment with rapamycin was determined by western blotting (lower). Data are representative of three experiments.

c, d) S6K phosphorylation of WT and *Lamtor1*$^{-/-}$ splenic DCs (**c, left**) and BMDCs (**d**) were determined by western blotting. Data are representative of three experiments. Quantification of p-S6K bands relative to S6K (**c,** right) (n=3). Statistical analysis was performed by Student's t-test (**a-c**) [means ± s.d.]

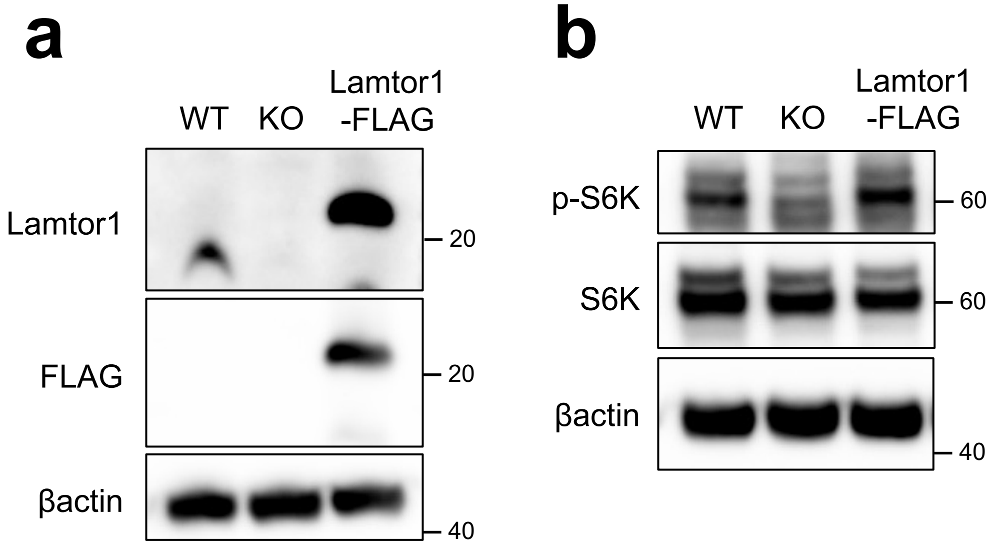

Supplementary Figure 4. Establishment of Lamtor1-KO-THP1 and Lamtor1-KO-Full-THP1

a, b) Generation of Lamtor1-KO-THP1 using the CRISPR/Cas9 system and re-expression of FLAG-tagged full-length Lamtor1 in Lamtor1-KO-THP1 by lentiviral transduction. Expression of Lamtor1 in parental THP1 (WT), Lamtor1-KO-THP1 (KO), and Lamtor1-KO-Full-THP1 was evaluated using anti-Lamtor1 and anti-FLAG antibodies (**a**). Phosphorylation of S6K in these cells was evaluated by western blotting using anti-phospho-S6K and anti-S6K antibodies (**b**). Data are representative of three experiments.

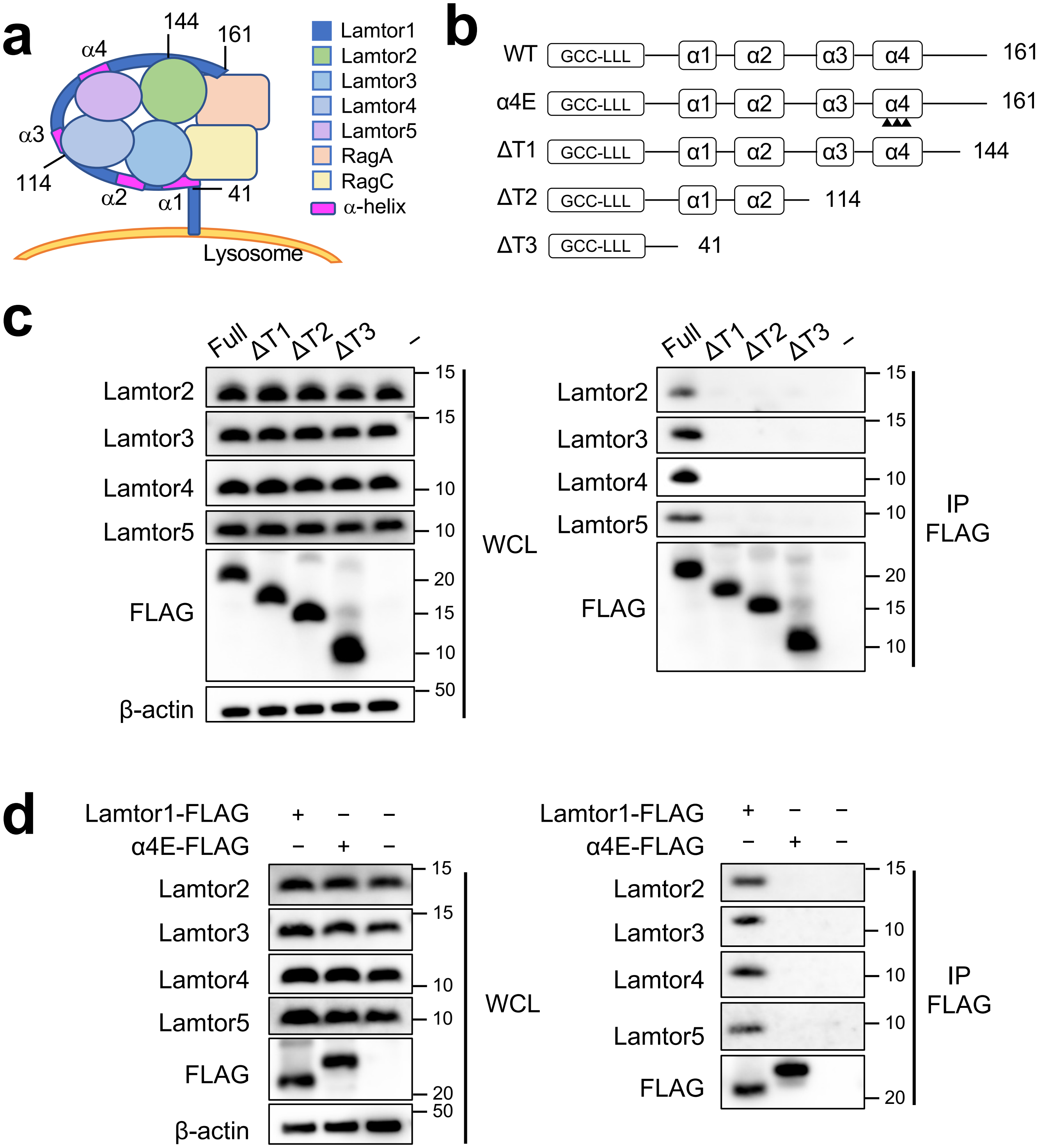

Supplementary Figure 5. Intact Lamtor1 is necessary for formation of the Ragulator complex.

a) Schematic of the Ragulator complex.

b) Schematic of mutant Lamtor1 (α4E, ΔT1, ΔT2, and ΔT3). Full length Lamtor1 is Met1–Pro161. Arrowheads indicate the position of amino acid substitutions (Leu129Glu, Ile135Glu, and Leu143Glu in the α4 helix). ΔT1 is Met1–Ser144, DT2 is Met1–Gln114, and DT3 is Met1–His41.

c, d) A mutant of Lamtor1 did not bind to Lamtor2–5. HEK293T cells transfected with FLAG-tagged full-length Lamtor1, FLAG-tagged truncated Lamtor1 (ΔT1, ΔT2, ΔT3) **(c),** or the FLAG-tagged substitution form of Lamtor1 (α4E) **(d),** was lysed and immunoprecipitated with anti-FLAG antibodies. Lamtor2–5 in whole-cell lysate (left) and precipitates (right) were detected by western blotting using antibodies against Lamtor2–5. Data are representative of three experiments.

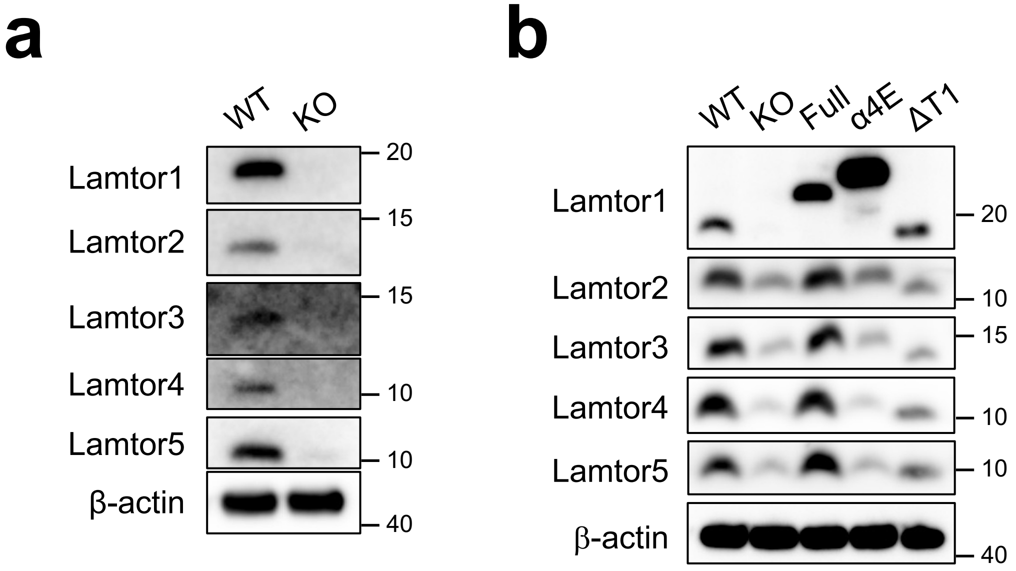

Supplementary Figure 6. Ragulator complex formation is defective in cells lacking Lamtor1 or expressing mutant Lamtor1.

a) Expression of Lamtor1–5 in WT and *Lamtor1*$^{-/-}$ BMDCs. Whole-cell lysates were prepared from WT and *Lamtor1*$^{-/-}$ BMDCs, and Lamtor1–5 were detected by western blotting. Data are representative of three experiments.

b) Expression of Lamtor1–5 in parental THP1 (WT), Lamtor1-KO-THP1 (KO), Lamtor1-KO-Full-THP1 (Full), Lamtor1-KO-α4E-THP1 (α4E), and Lamtor1-KO-ΔT1-THP1 (ΔT1). Whole-cell lysates were obtained from these cells, and Lamtor1–5 were detected by western blotting. Data are representative of three experiments.

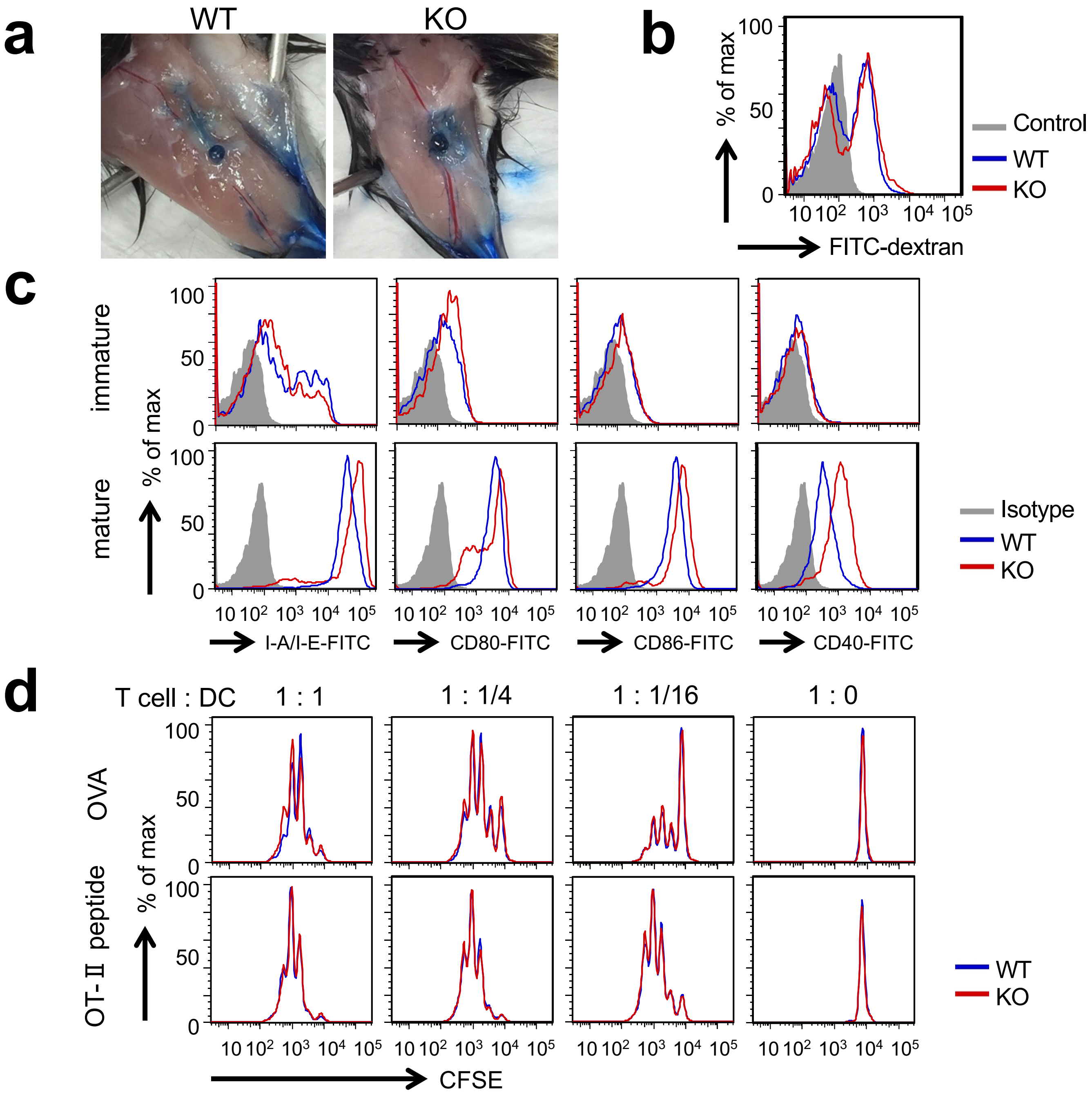

Supplementary Figure 7. Characteristics of *Lamtor1*$^{-/-}$ DCs

a) Staining of afferent lymphatics and popliteal LNs. One hour after injection of Evans blue dye (5 mg/ml) into the footpad of WT (upper) and CD11c–*Lamtor1*$^{-/-}$ (lower) mice, afferent lymphatics and popliteal LNs were observed.

b) FITC-dextran uptake of DCs. WT (blue line) and *Lamtor1*$^{-/-}$ BMDCs (red line) were incubated with FITC-dextran at 37 °C for 30 min. Control cells (gray) were cultured on ice with FITC-dextran.

c) Expression of co-stimulatory molecules in WT and *Lamtor1*$^{-/-}$ DCs. WT (blue line) or *Lamtor1*$^{-/-}$ DCs (red line) were stimulated with or without LPS (200 ng/ml) for 18 h, and the levels of co-stimulatory molecules (I-A/I-E, CD80, CD86, CD40) were assessed by FACS.

d) *In vitro* OT-II T-cell proliferation by OVA- or OT-II peptide–pulsed WT and *Lamtor1*$^{-/-}$ DCs. CFSE-labeled $CD4^{+}$ OT-II T cells were cultured with WT (blue line) or *Lamtor1*$^{-/-}$ BMDCs (red line) in the presence of OVA protein (upper) or OT-II peptide (lower). CFSE dilution was assessed by FACS.

Data are representative of three experiments.

Supplemental Tables

Identified Proteins	Accession Number	Molecular Weight	total spectrum count WT	total spectrum count KO
Supervillin	E9Q3Z5	230 kDa	6.1419	0
Putative uncharacterized protein	Q3ULB1 (+1)	47 kDa	6.1419	0
LIM domain and actin-binding protein 1	Q9ERG0	84 kDa	6.1419	0
T-complex protein 1 subunit beta	P80314	57 kDa	4.0946	0
Phostensin	Q8BQ30	66 kDa	4.0946	0
Cytochrome b-c1 complex subunit 2, mitochondrial	Q9DB77	48 kDa	4.0946	0
Ragulator complex protein LAMTOR2	Q9JHS3	13 kDa	4.0946	0
TAR DNA-binding protein 43	A0A087WRZ5 (+5)	32 kDa	3.071	0
Serine/threonine-protein phosphatase	A0A0G2JGC1 (+2)	31 kDa	3.071	0
CAD protein	B2RQC6 (+2)	243 kDa	3.071	0
Coronin-1A	O89053	51 kDa	3.071	0
Annexin A2	P07356	39 kDa	3.071	0
Dynactin subunit 4	Q8CBY8	53 kDa	3.071	0
Chloride intracellular channel protein 1	Q9Z1Q5	27 kDa	3.071	0
Ubiquitin-like modifier-activating enzyme ATG7	A0A0A0MQN4 (+3)	82 kDa	2.0473	0
Voltage-gated potassium channel subunit beta-2	E0CXZ9 (+2)	43 kDa	2.0473	0
Proteasome activator complex subunit 2	E0CZ90 (+2)	19 kDa	2.0473	0
Puromycin-sensitive aminopeptidase	F6QYF8 (+1)	99 kDa	2.0473	0
Myosin phosphatase Rho-interacting protein	F6RND9 (+3)	114 kDa	2.0473	0
Voltage-dependent anion-selective channel protein 2	G3UX26 (+1)	30 kDa	2.0473	0
Heterogeneous nuclear ribonucleoprotein U	G3XA10 (+1)	87 kDa	2.0473	0
Pro-interleukin-16	O54824	141 kDa	2.0473	0
Ras-related protein Rab-7a	P51150	23 kDa	2.0473	0
40S ribosomal protein S11	P62281	18 kDa	2.0473	0
40S ribosomal protein S13	P62301 (+1)	17 kDa	2.0473	0
Sorting and assembly machinery component 50 homolog	Q8BGH2	52 kDa	2.0473	0
Phosphatidylinositol 4-kinase alpha	A0A140T8I9 (+1)	231 kDa	2.0473	0
60S ribosomal protein L37a	P61514	10 kDa	2.0473	0
Nuclear pore complex protein Nup160	Q9Z0W3	158 kDa	2.0473	0
Heat shock protein 75 kDa, mitochondrial	Q9CQN1	80 kDa	2.0473	0
Histone H2A	A0A0N4SV66 (+10)	14 kDa	2.0473	0
Glutamate-cysteine ligase regulatory subunit	A0A0G2JDI4 (+1)	11 kDa	2.0473	0
Elongation factor 1-beta	G3UX43 (+2)	8 kDa	2.0473	0
40S ribosomal protein S23	P62267	16 kDa	2.0473	0
Glycine--tRNA ligase	Q9CZD3	82 kDa	2.0473	0
Protein S100-A6	P14069	10 kDa	2.0473	0
Protein Rpl23a-ps3	A0A140T8M7 (+1)	18 kDa	2.0473	0

Supplementary Table 1. Candidate Lamtor1-interacting proteins, as determined by mass spectrometry analysis.

WT and *Lamtor1*$^{-/-}$ DCs were lysed and immunoprecipitated using anti-Lamtor1 antibody. After elution of lysates, LC-MS/MS was performed. Validation of MS/MS-based peptide and protein identification was performed using the Scaffold software (ver. 4.4.3). Among 584 proteins that were identified, proteins with a total spectrum count of 0 in the knockout and a total spectrum count above 2 in WT were selected.

Reagents		
Name	Vender	Catalog number
LPS	Sigma	
PMA	Sigma	
FITC-isomer I	Sigma	
Rapamycin	Sigma	
fibronectin	Sigma	
collagenase D	Roche	
type I collagen	BD Biosciences	
Calcein-AM	Invitrogen	
CFSE	Invitrogen	
phalloidin-546	Invitrogen	
AcidiFluor-ORANGE	GORYO Chemistry	GC301
Torin1	Sellec	S2827
U0126	Calbiochem	662009
(-)-Blebbistatin	Cayman Chemical	13013
recombinant mouse CCL19	R & D systems	
recombinant mouse CCL21	R & D systems	
recombinant human MCP-1	R & D systems	
mouse GM-CSF	Wako	
Evans blue	Nacalai tesque	
MISSION shRNA plasmid for MPRIP	Sigma	TRCN0000048648

Antibodies for immunohistochemistry			
Name	Clone	Vender	Catalog number
Phospho-Myosin Light Chain 2 (Ser19) Antibody		Cell Signaling Technology	#3671
Anti-MPRIP antibody		Sigma	HPA022901
Anti-LAMP1 antibody	1D4B	Abcam	ab25245
Anti-MYPT1 antibody		Santa Cruz	sc-514261
Alexa Fluor® 647 anti-mouse CD11c Antibody	N418	Biolegend	117312
anti-PECAM-1 antibody		Abcam	ab28364
Monoclonal ANTI-FLAG® M2 (mouse)	M2	Sigma	F3165

Antibodies for flow cytometry			
Name	Clone	Vender	Catalog number
APC anti-mouse CD11c antibody	N418	Biolegend	117310
APC anti-mouse I-A/I-E Antibody	M5/114.15.2	Biolegend	107614
FITC anti-mouse I-A/I-E Antibody	M5/114.15.2	Biolegend	107605
Brilliant Violet 421™ anti-mouse I-A/I-E Antibody	M5/114.15.2	Biolegend	107632
APC anti-mouse CD197 (CCR7) Antibody	4B-12	Biolegend	120108
APC anti-mouse/human CD45R/B220 Antibody	RA3-6B2	Biolegend	103212
APC anti-mouse CD3ε Antibody	145-2C11	Biolegend	100312
FITC anti-mouse CD8a Antibody	53-6.7	Biolegend	100705
PE/Cy7 anti-mouse CD8a Antibody	53-6.7	Biolegend	100722
FITC anti-mouse CD45 Antibody	30-F11	Biolegend	103107
APC anti-mouse CD317 (BST2, PDCA-1) Antibody	927	Biolegend	127016
FITC anti-mouse CD86 Antibody	GL1	Biolegend	105005
Brilliant Violet 421 anti-Mouse CD11b Antibody	M1/70	Biolegend	101235
FITC anti-Mouse Ly6C Antibody	HK1.4	Biolegend	128005
APC-Cy7 Anti-Mouse Ly6G Antibody	1A8	Biolegend	127623
PE anti-Mouse CD103 Antibody	2E7	eBiolegend	121405

Antibodies for flow cytometry

Name	Clone	Vender	Catalog number
PE Anti-Mouse CD11c Antibody	HL3	BD pharmingen	557401
FITC Anti-Mouse CD80 Antibody	16-10A1	BD pharmingen	561954
FITC Anti-Mouse CD40 Antibody	3/23	BD pharmingen	553790
PE Anti-Mouse NK1.1 Antibody	PK136	eBioscience	12-5941-82
FITC Anti-Mouse CD4 Antibody	GK1.5	eBioscience	11-0041-82

Antibodies for western blotting and immunoprecipitation

Name	clone	Vender	Catalog number
Myosin Light Chain 2 Antibody		Cell Signaling Technology	#3672
Phospho-Myosin Light Chain 2 (Ser19) Antibody		Cell Signaling Technology	#3671
p70 S6 Kinase Rabbit mAb	49D7	Cell Signaling Technology	#2708
Phospho-p70 S6 Kinase (Thr389) Rabbit mAb	108D2	Cell Signaling Technology	#9234
β-Actin Rabbit mAb (HRP Conjugate)	13E5	Cell Signaling Technology	#5125
MYPT1 Antibody		Cell Signaling Technology	#2634
Phospho-MYPT1 (Thr696) Antibody		Cell Signaling Technology	#5163
M-RIP Rabbit mAb	D8G8R	Cell Signaling Technology	#14396
LAMTOR1/C11orf59 XP® Rabbit mAb	D11H6	Cell Signaling Technology	#8975
LAMTOR2/ROBLD3 Rabbit mAb	D7C10	Cell Signaling Technology	#8145
LAMTOR3/MAPKSP1 Rabbit mAb	D38G5	Cell Signaling Technology	#8168
LAMTOR4/C7orf59 Rabbit mAb	D6A4V	Cell Signaling Technology	#12284
LAMTOR5/HBXIP Rabbit mAb	D4V4S	Cell Signaling Technology	#14633
GFP Antibody		Cell Signaling Technology	#2555
ANTI-FLAG® M2-Peroxidase (HRP) mAb		Sigma	A8592
V5 Tag Monoclonal Antibody, HRP		Invitrogen	R96125
HA-tag monoclonal antibody, HRP	Y-11	Santa Cruz	Sc-805
Purified anti-CRISPR (CAS9) Antibody	7A9	BioLegend	844301
DYKDDDDK Tag Antibody		Cell Signaling Technology	2368
V5 Tag Monoclonal Antibody		Invitrogen	R960-25
Anti-MPRIP antibody		Sigma	HPA022901

Primers for generation of vectors and KO-cell-line

Generation of Lamtor1-KO-THP1	5’-TGCGAGCGGAAGGCAGGCTG-3’
	5’-GCTCCGGGACAGGGGGTACG-3’
	5’-TCCGGTCACATGACCCGCGG-3’
	5’-CTGCTACAGCAGCGAGAACG-3’
	5’-GGCCTGCTCATCAGTGCGAG-3’
	5’-AGGTGCTCACCTGTACTGCC-3’
p18_Kozak_Fw (Attaches NotI site)	5’-AATTCTGCAGCGGCCGCGCCACCATGGGGTGCTGC-3'
p18_FLAG_Rv	5’-GGAGAGGGGCGGATCCTCACTACTTGTCATCGTCAT-3'
p18_trunc1_Rv	5’-CCGTCATGGTCTTTGTAGTCAGAAAGTGCACTATAGGCAT-3'
p18_trunc3_Rv	5’-CCGTCATGGTCTTTGTAGTCCTGGCTGGTGAGAGATGGCA-3'
p18_trunc5_Rv	5’-CCGTCATGGTCTTTGTAGTCATGGTAGTTGGGCTCGGCTC-3'
p18_mutant_Rv	5’-ATCACCGTCATGGTCTTTGTAGTCTGGGATCCCAAACTGTACAACCAG-3'
3xFLAG_oligo_Rv	5’-TCACTACTTGTCATCGTCATCCTTGTAGTCGATGTCATGATCTTTATAATCACCGTCATGGTCTTTGTAGTC-3'
p18_FLAG_Rv (Attaches BamHI site, same as mLAMTOR1_3XFLAG_intoVenus_Rv)	5’-GGAGAGGGGCGGATCCTCACTACTTGTCATCGTCAT-3'

		PCR1: Cloning Lamtor1		PCR2: Ligation of 3xFlag		
Label in Note	Label in Paper	Fw	Rv	Fw	Rv1	Rv2
Full (for attaching Kozak)	Full	p18_Kozak_Fw	p18_FLAG_Rv	p18_Kozak_Fw	3xFLAG_oligo_Rv	p18_FLAG_Rv
Trunc 1	ΔT1	p18_Kozak_Fw	p18_trunc1_Rv	p18_Kozak_Fw	3xFLAG_oligo_Rv	p18_FLAG_Rv
Trunc 3	ΔT2	p18_Kozak_Fw	p18_trunc3_Rv	p18_Kozak_Fw	3xFLAG_oligo_Rv	p18_FLAG_Rv
Trunc 5	ΔT3	p18_Kozak_Fw	p18_trunc5_Rv	p18_Kozak_Fw	3xFLAG_oligo_Rv	p18_FLAG_Rv
a4E	α4E	p18_Kozak_Fw	p18_mutant_Rv	p18_Kozak_Fw	3xFLAG_oligo_Rv	p18_FLAG_Rv

**Primers for mice screening**	
Lamtor1^{flox} and Lamtor1 alleles	5’-AAGGATTCGGAGTTAGAGACTAGGAC-3’
	5’-TGAGGATTCGAGTGGTGAGATACGA-3’
CD11c-Cre-Tg mice	5’-AGACTCAGCTCAAGTGCTAC-3’
	5’-GCGAACATCTTCAGGTTCTG-3’
LysM-Cre-Tg mice	5’-CTTGCTGTGTGTTGTTCTGTGCTGAGG-3’
	5’- GCATAACCAGTGAAACAGCATTGC-3’

Supplementary Table 2. Reagents, antibodies, and primers

Reagents, antibodies, and primers used in this study are listed.
