# Supplementary material for: The lysosomal Ragulator complex plays an essential role in leukocyte trafficking by activating myosin II
Source: Nat Commun. 2021 Jun 7;12:3333. doi: 10.1038/s41467-021-23654-3 (PMC8184920; doi:10.1038/s41467-021-23654-3)
Supplement: Supplementary file 3 — Description of Additional Supplementary Files [file 41467_2021_23654_MOESM3_ESM.pdf]

Description of Additional Supplementary Files

File Name: Supplementary Movie 1
Description: **Localization of lysosomes during DC migration in a confined 3D environment.** BMDCs were labeled with 1 mM AcidiFluor ORANGE and stimulated with LPS for 2 h. Movement of DCs and AcidiFluor-positive lysosomes (magenta) in response to CCL19 (5µg/ml) in type I collagen gel (2 mg/ml) in a Zigmond chamber was observed at 1-min intervals by time-lapse video imaging.

File Name: Supplementary Movie 2
Description: **Directional migration of WT DCs in response to CCL19 in a 2D environment.** Horizontal migration of WT BMDCs in response to CCL19 (5 µg/ml) in an EZTAXIScan device (slit size, 5 µm) was visualized at 1-min intervals by time- lapse video imaging

File Name: Supplementary Movie 3
Description: **Directional migration of Lamtor1–/– DCs in response to CCL19 in a 2D environment.** Horizontal migration of Lamtor1–/– BMDCs in response to CCL19 (5 µg/ml) in an EZ-TAXIScan device (slit size, 5 µm) was visualized at 1-min intervals by time-lapse video imaging.

File Name: Supplementary Movie 4
Description: **Motility of WT DCs in response to CCL19 in 3D collagen matrices.** Movement of WT BMDCs in response to CCL19 (5 µg/ml) in type I collagen gels (2 mg/ml) in a Zigmond chamber was observed at 1-min intervals by time-lapse video imaging

File Name: Supplementary Movie 5
Description: **Motility of Lamtor1–/– DCs in response to CCL19 in 3D collagen matrices.** Movement of Lamtor1–/– BMDCs in response to CCL19 (5 µg/ml) in type I collagen gels (2 mg/ml) in a Zigmond chamber was observed at 1-min intervals by time-lapse video imaging
